# Mutation of Hydrophobic Residues in the C-Terminal Domain of the Marburg Virus Matrix Protein VP40 Disrupts Trafficking to the Plasma Membrane

**DOI:** 10.3390/v12040482

**Published:** 2020-04-24

**Authors:** Kaveesha J. Wijesinghe, Luke McVeigh, Monica L. Husby, Nisha Bhattarai, Jia Ma, Bernard S. Gerstman, Prem P. Chapagain, Robert V. Stahelin

**Affiliations:** 1 Department of Chemistry and Biochemistry, University of Notre Dame, Notre Dame, IN 46556, USA; kaveeshaw@gmail.com (K.J.W.); lgmcveig@iu.edu (L.M.); 2 Department of Medicinal Chemistry and Molecular Pharmacology and the Purdue Institute for Inflammation, Immunology and Infectious Disease, Purdue University, West Lafayette, IN 47907, USA; mhusby@purdue.edu; 3 Department of Physics, Florida International University, Miami, FL 33199, USA; nbhat006@fiu.edu (N.B.); gerstman@fiu.edu (B.S.G.); chapagap@fiu.edu (P.P.C.); 4 Bindley Bioscience Center, Purdue University, West Lafayette, IN 47907, USA; jm4757@columbia.edu; 5 Biomolecules Sciences Institute, Florida International University, Miami, FL 33199, USA

**Keywords:** ebola virus, filovirus, lipid binding, marburg virus, membrane trafficking, virus assembly, VP40

## Abstract

Marburg virus (MARV) is a lipid-enveloped negative sense single stranded RNA virus, which can cause a deadly hemorrhagic fever. MARV encodes seven proteins, including VP40 (mVP40), a matrix protein that interacts with the cytoplasmic leaflet of the host cell plasma membrane. VP40 traffics to the plasma membrane inner leaflet, where it assembles to facilitate the budding of viral particles. VP40 is a multifunctional protein that interacts with several host proteins and lipids to complete the viral replication cycle, but many of these host interactions remain unknown or are poorly characterized. In this study, we investigated the role of a hydrophobic loop region in the carboxy-terminal domain (CTD) of mVP40 that shares sequence similarity with the CTD of Ebola virus VP40 (eVP40). These conserved hydrophobic residues in eVP40 have been previously shown to be critical to plasma membrane localization and membrane insertion. An array of cellular experiments and confirmatory in vitro work strongly suggests proper orientation and hydrophobic residues (Phe^281^, Leu^283^, and Phe^286^) in the mVP40 CTD are critical to plasma membrane localization. In line with the different functions proposed for eVP40 and mVP40 CTD hydrophobic residues, molecular dynamics simulations demonstrate large flexibility of residues in the EBOV CTD whereas conserved mVP40 hydrophobic residues are more restricted in their flexibility. This study sheds further light on important amino acids and structural features in mVP40 required for its plasma membrane localization as well as differences in the functional role of CTD amino acids in eVP40 and mVP40.

## 1. Introduction

Marburg virus (MARV), a close relative of Ebola virus (EBOV), belongs to the *Filoviridae* family of viruses. EBOV and MARV cause hemorrhagic fever in humans and non-human primates, which can have high rates of fatality [1]. Filoviruses have a host plasma membrane-derived lipid envelope that gives rise to filamentous virions that can vary in their overall morphology (e.g., hooked, six-shaped or round) [2]. MARV has a negative sense RNA genome, which encodes seven proteins: a transmembrane glycoprotein (GP), the matrix protein VP40 (mVP40), and several proteins that make up the nucleocapsid (NC) including nucleoprotein (NP), VP24, VP30, VP35 and the polymerase L [3]. 

VP40 is a peripheral membrane protein that coats the inner leaflet of the viral lipid envelope forming the viral matrix layer, which connects the viral NC with the lipid envelope. Inside the infected host cells, both EBOV VP40 (eVP40) and mVP40 facilitate the assembly and budding of nascent virions. Expression of either eVP40 or mVP40 in mammalian cells, in the absence of other filovirus proteins, led to the formation of filamentous virus-like particles (VLPs) that resemble the authentic virions [4,5,6,7]. eVP40 and mVP40 form dimers [8,9] using an amino-terminal domain (NTD) α-helical interface, where the dimers are thought to be building blocks for large VP40 oligomers that form at the plasma membrane and are necessary for budding [8,9,10,11,12,13,14]. Notably, mutation of the dimer interface of eVP40 or mVP40 abrogated VLP formation and significantly reduced VP40 plasma membrane localization [8,9,14]. Though in general, eVP40 and mVP40 are thought to assemble at the plasma membrane inner leaflet in a similar capacity, fundamental differences in their membrane binding properties [8,9,10,11,12,14] as well as trafficking pathways to the plasma membrane [15,16] have been identified. These differences in eVP40 and mVP40 interactions with the host-cell may stem from differences in their amino acid sequences as mVP40 and eVP40 harbor 34% amino acid sequence identity with the majority of sequence conservation observed in the (NTD) of these proteins [8]. The VP40 carboxy-terminal domain (CTD), which contains the membrane interaction domain or the “basic patch” has only 15% sequence identity. 

**Figure 1:**
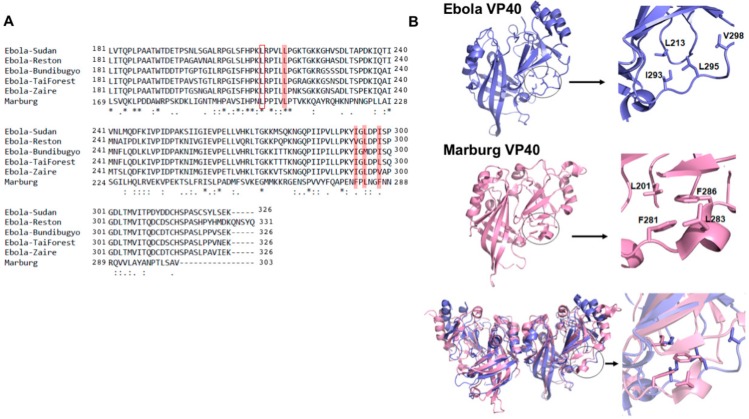
Marburg virus (MARV) VP40 (mVP40) and Ebola virus (EBOV) VP40 (eVP40) proteins have a similar hydrophobic loop region in the C-terminal domain (CTD). (**A**) Sequence alignment of eVP40 and mVP40 shows that Leu^201^ of mVP40 is conserved and aligns with Leu^213^ of eVP40 (open red box). The hydrophobic loop region of eVP40 Zaire composed of Ile^293^, Leu^295^ and Val^298^ residues aligns with Phe^281^, Leu^283^ and Phe^286^ residues of mVP40 indicating conservation of hydrophobic character of this region (solid red box). The sequence alignment was created using clustalW. “*” shows conserved residues in all 5 strains of eVP40 and mVP40. “**:**” Represents residues that are mostly conserved “.“ represents residues that are somewhat conserved between the strains. (**B**) Orientation of hydrophobic loop region on eVP40 and mVP40. eVP40 monomer (blue) and mVP40 monomer (pink) with the hydrophobic loop enlarged. The bottom panel is a structural alignment of the eVP40 and mVP40 proteins is also shown.

Previous studies have demonstrated that interaction of mVP40 with the plasma membrane depends on electrostatic interactions between mVP40 basic residues and the anionic charge of lipids such as phosphatidylserine (PS) and PI(4,5)P_2_. mVP40 interacts with a broad range of anionic phospholipids functioning as an anionic charge sensor [8,10,12]. In contrast, eVP40 primarily interacts with the plasma membrane displaying selectivity for phosphatidylserine (PS) [9,10,14,17] and phosphatidylinositol-4,5-bisphosphate (PI(4,5)P_2_) [18]. Further, eVP40 was also shown to penetrate membranes containing PS using a hydrophobic loop region at the (CTD) supplementing electrostatic interactions during membrane association [19,20]. In contrast, mVP40 did not significantly insert into the plasma membrane to form hydrophobic interactions with the membrane hydrocarbon region [12]. The hydrophobic loop region on eVP40 that inserts into the plasma membrane was composed of Ile^293^, Leu^295^ and Val^298^ and these residues are at the same interface as the Leu^213^ residues that is conserved in eVP40 [19,20]. The Leu^213^ residue of eVP40 was shown to disrupt VP40 plasma membrane localization and budding when mutated [21] and the authors indicated the importance of Leu^213^ and surrounding residues for the structure and/or self-oligomerization. In support of this hypothesis, a L213A mutation was shown to reduce eVP40 oligomerization in human cells [19]. Leu^213^ of eVP40 aligns with Leu^201^ in mVP40 and may also be important as a bridging region between the CTD and NTD (Figure 1). Hydrophobic residues at positions 293, 295, and 298 in eVP40 that were shown to be important for plasma membrane localization and oligomerization of VP40 align with hydrophobic residues (Phe^281^, Leu^283^, and Phe^286^) in mVP40.

eVP40 and mVP40 have also been shown to harbor some differences in their plasma membrane trafficking pathways. For instance, mVP40 was shown to interact with cellular membranes following its synthesis inside the cytoplasm of infected cells and later accumulated in multivesicular bodies [22,23]. Subsequently, mVP40 then appears in membrane clusters beneath the plasma membrane before finally localizing to the plasma membrane inner leaflet [8,10,22]. Therefore, mVP40 has been hypothesized to exploit the retrograde endosomal pathway to reach the plasma membrane. In contrast, eVP40 has been shown to interact with the Sec24C protein, which is a component of the COPII transport machinery, which normally traffics proteins between the ER and Golgi [16]. eVP40 has been hypothesized to hijack and perhaps redirect the COPII vesicle transport system to achieve plasma membrane localization [16]. 

To examine the role of hydrophobic residues in mVP40 that are conserved with critically important hydrophobic residues in the eVP40 CTD, we prepared single, double, and triple mutations of several residues in the mVP40 CTD (Figure 1). To determine the role of these amino acids in mVP40 trafficking and assembly, we used confocal microscopy and transmission electron microscopy (TEM) to examine mVP40 localization and virus-like particle (VLP) formation. Complementary in vitro studies were performed for protein quality control using a lipid-binding assay and analytical ultracentrifugation to assess mVP40 dimerization. Lastly, molecular dynamics simulations were undertaken to examine the intramolecular interactions of eVP40 and mVP40 at these CTD hydrophobic residues. Taken together, our results demonstrate the importance of hydrophobic residues at positions 281, 283, and 286 in mVP40 for the proper plasma membrane localization and assembly of mVP40.

## 2. Materials and Methods 

Lipids, 1-palmitoyl-2-oleoyl-*sn*-glycero-3-phosphocholine (POPC), 1-palmitoyl-2-oleoyl-*sn*-glycero-3-phospho-L-serine (POPS), 1-palmitoyl-2-oleoyl-*sn*-glycero-3-phosphoethanolamine (POPE), and 1,2-dioleoyl-*sn*-glycero-3-phospho-(1′-myo-inositol-monophosphate) (PI3P) were from Avanti Polar Lipids, Inc. (Alabaster, AL, USA) and used without further purification. Complete Mini-EDTA-free protease inhibitor cocktail was from Roche (Indianapolis, IN, USA), phenylmethylsulfonylfluoride (PMSF) and bicinchoninic acid (BCA) protein assay kit were from Thermo Fisher Scientific (Waltham, MA, USA). Lipofectamine 2000, Lipofectamine LTX and PLUS reagent were from Life Technologies (Grand Island, NY, USA). Ni-NTA agarose was from Qiagen (Valencia, CA, USA). Anti c-Myc monoclonal antibody (9E10) was from Thermo Fisher Scientific (Waltham, MA, USA), anti-EGFP antibody (F56-6A1.2.3) was from Invitrogen (Waltham, MA, USA), and sheep polyclonal antibody to mouse IgG-HRP conjugate was from Abcam (Cambridge, England). His_6_-mVP40 in pET46 was a kind gift from Dr. Erica Ollmann Saphire (La Jolla Institute for Immunology, La Jolla, CA, USA). 

Q5-site directed mutagenesis kit was from New England Biolabs Inc. (Ipswich, MA, USA). Primers were designed for mVP40 mutants and synthesized by Integrated DNA Technologies (Coralville, Iowa, USA). The following primers were used: L201A 5′-CCACCCGAATGCTCCGCCTATTGTTCTACCAAC-3′ (forward primer), 5′-ATGGAGACTGCGGGATGC-3′ (reverse primer), F281A 5′-ACCTGAGAACGCTCCTTT GAATGGCTTC-3′ (forward primer), 5′-GCTTGAAAATAAACCACGG-3′ (reverse primer) L283A 5′-GAACTTCCCTGCTAATGGCTTCAATAAC-3′ (forward primer), 5′-TCAGGTGCTTGAAAATA AAC-3′ (reverse primer), F286A 5′-TTTGAATGGCGCTAATAACAGACAAGTTGTGC-3′ (forward primer), 5′-GGGAAGTTCTCAGGTGCTTG-3′ (reverse primer), F281A/F286A 5′-TTGAATGGCGC TAATAACAGACAAGTTGTGCTAG-3′ (forward primer), 5′-GGAGCGTTCTCAGGTGCT-3′ (reverse primer), F281A/L283A/F286A 5′-TGCTAATGGCGCTAATAACAGAC-3′ (forward primer), 5′-GGAGCGTTCTCAGGTGCTTGAAAATAAAC-3′ (reverse primer). 

### 2.1. Site Directed Mutagenesis

Site directed mutagenesis on mVP40 was performed as directed by the manufacturer instructions using the above listed primers.

### 2.2. Cell Culture

COS-7 and HEK293 cells (from American Type Culture Collection, Manassas, VA, USA) were maintained in Dulbecco’s Modified Eagle’s Medium (DMEM) (Invitrogen, Carlsbad, CA, USA) containing 10% FBS and 1% Penstrep at 37 °C in a 5% CO_2_ humidified incubator. Cells were grown to 70–80% confluency before passaging or transfection. Transfections were performed using Lipofectamine 2000 reagent for COS-7 cells and Lipofectamine LTX and Plus reagent for HEK293 cells according to the manufacturer’s protocols. Both COS-7 and HEK293 cells have previously been used for research examining VP40 trafficking, localization and budding from the plasma membrane [10,11,12,13,14].

### 2.3. Heterologous Expression Test

COS-7 cells transfected with EGFP tagged mVP40 or mutants were collected 14 h post transfection. Cells were lysed using RIPA buffer (10 mM Tris-HCl, pH 8.0 containing 150 mM NaCl, 2 mM EDTA, 0.5% sodium deoxycholate, 0.1% SDS, 1% Triton-X100, and with PMSF and protease inhibitor cocktail added just before use) by incubating on ice for 1 h while briefly vortexing every 15 min. The cell lysate was then centrifuged at 25,000× *g* for 17 min at 4 °C. Supernatants were collected and assessed by western blot analysis using anti-EGFP antibody or anti-mVP40 antibody with anti GAPDH antibody employed for protein loading controls. 

### 2.4. Confocal Imaging and Analysis

Routine imaging of all cell lines was performed on a Zeiss LSM 710 (Carl Zeiss Microscopy, Jena, Germany) inverted microscope using a Plan Apochromat 63× 1.4 numerical aperture oil objective. EGFP was excited using the 488 nm line of the Ar ion laser with the emission collected through a 493–550 nm filter. Unless otherwise stated, cells were imaged 14 h post-transfection. Image J software was used to assess the plasma membrane and cytoplasmic intensities to calculate the percentage of plasma membrane localization as described in Wijesinghe, K.J. et al. (2016) [8]. An example of the plasma membrane stenciling process is shown in Figure S1. The plasma membrane stenciling method was compared with the plasma membrane localization of EGFP-mVP40 using the plasma membrane marker, wheat germ agglutin (WGA)-Alexa Fluor 647 (Figure S1). To further demonstrate the selectivity in plasma membrane localization of mVP40, the monomeric mutant T105R was used, which abrogated plasma membrane localization (Figure S1).

### 2.5. Transmission Electron Microscopy

Cells were fixed in 2.5% glutaraldehyde in 0.1 M sodium cacodylate buffer, rinsed, and embedded in agarose. Small pieces of cell pellet were post-fixed in buffered 1% osmium tetroxide containing 0.8% potassium ferricyanide and en bloc stained in 1% uranyl acetate. They were then dehydrated with a graded series of ethanol, transferred into acetonitrile and embedded in EMbed-812 resin. Thin sections were cut on a Reichert-Jung Ultracut E ultramicrotome (Reichert-Jung, Wien, Austria) and stained with 4% uranyl acetate and lead citrate. Images were acquired on a FEI Tecnai T12 electron microscope (Field Electron and Ion Company, Hillsboro, OR, USA) equipped with a tungsten source and operating at 80 kV. In order to assess the quantitative changes in pre-VLP formation or WT and mutations, the area of each cell was measured and the number of pre-VLPs (*only counted VLPs visibly connected to the plasma membrane surface) were counted. Values are reported as mean ± SD. Brown-Forsythe and Welch ANOVA was then performed with multiple comparisons (compared to WT mVP40).

### 2.6. Protein Expression and Purification

mVP40 protein and L201A harboring a 6x-His Tag were expressed and purified as described in Wijesinghe K.J., et al. (2016) [10].

### 2.7. Multilamellar Vesicle Sedimentation Assay

A multilamellar vesicle (MLV) sedimentation assay was performed as previously described in in Wijesinghe K.J., et al. (2016) [10] and Oda S., et al (2016) [8]. 

### 2.8. Sedimentation Velocity Measurements

Sedimentation velocity measurements were performed on a ProteomeLab XL-L (Beckman Coulter, Brea, CA, USA) analytical ultracentrifuge. Double-sector analytical ultracentrifugation sample cells were assembled with a 12-mm epon-charcoal centerpiece and sapphire windows. The 400 µL purified protein in HEPES buffer (pH 7.4) and the same volume of HEPES buffer (pH 7.4) as bland reference were loaded to each sector of a sample cell. The sample cells were placed in a Ti-60 rotor, which was then loaded in the centrifuge rotor chamber and equilibrated at 20 °C for two hours under the vacuum before centrifugation. The centrifugation was carried out at 50,000 rpm at 20 °C. The progress of centrifugation was monitored by recording the absorbance at 280 nm. The sedimentation velocity boundaries were analyzed by the c(s) model in SEDFIT version 15.01b [24] (by Peter Schuck, NIH/NIBIB, Bethesda, MD, USA) and c(s) plots were generated with GUSSI version 1.1.0 [25] (University of Cambridge, Cambridge, UK).

### 2.9. Molecular Dynamics Simulations

Structures of the EBOV and MARV VP40 dimers were obtained from the Protein Data Bank (PDB ids 4LDB and 5B0V, respectively) [8,9]. The missing residues were modeled using Modeller software (University of California San Francisco, San Francisco, CA, USA) [26]. All-atom molecular dynamics simulations were performed using NAMD 2.12 (University of Illinois at Urbana- Champaign, Urbana and Champaign, IL, USA) [27] with Charmm36m (University of Illinois at Urbana- Champaign, Urbana and Champaign, IL, USA) [28] force field. Each system was solvated with TIP3 water molecules and neutralized with counter-ions. The simulations were performed with periodic boundary conditions. The long range electrostatic interactions were treated with the particle mesh Ewald (PME) [29] method and the covalent bonds involving hydrogen atoms were treated as rigid bonds using the SHAKE algorithm [30]. The simulations were carried out at 300 K, which was maintained using Langevin thermostat. Similarly, pressure was constrained with Nose-Hoover Langevin-piston method [31] with a piston period of 50 fs and decay of 25 fs. Each system was minimized for 10,000 steps and equilibrated for 100 ps using 1 fs time-step. The production runs were performed with 2 fs time-step for 200 ns. Visual molecular dynamics (VMD) [32] was used to visualize and analyze the trajectories.

## 3. Results

### 3.1. A Hydrophobic Loop in the VP40 CTD is Conserved Across eVP40 and mVP40

From sequence alignment and (Figure 1A) aligning of crystal structures of eVP40 and mVP40, we observed that mVP40 also has a hydrophobic loop in the CTD showing similar orientation to the eVP40 hydrophobic loop (Figure 1A,B). This region on mVP40 is composed of Phe^281^, Leu^283^ and Phe^286^ and therefore shows conservation of hydrophobic character. The orientation of the hydrophobic loop of mVP40 is further away from the basic patch involved in binding membranes and adjacent to a more distant hydrophobic residue, Leu^201^ (Figure 1B). Therefore, due to the hydrophobic residue orientation in mVP40, it is less likely to insert into the membrane like the eVP40 hydrophobic loop, further explaining our previous experimental observations [12,19,20]. The mVP40 hydrophobic loop is also not completely unstructured as residues 282–286 and forms a short α-helical turn in contrast to the unstructured region of residues (293–298) in eVP40. We hypothesized the conservation of hydrophobic character in this CTD region, despite difference in positioning and secondary structure with eVP40, may play an important role in mVP40 plasma membrane trafficking or assembly and budding. 

### 3.2. Cellular Imaging of mVP40 Hydrophobic Mutants Reveals a Stalled Vesicle Morphology

**Figure 2:**
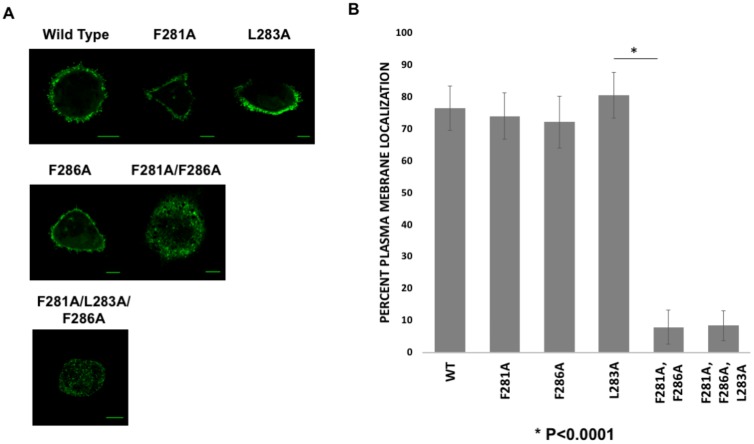
Cellular localization of WT mVP40 and hydrophobic residue mutants. (**A**) COS-7 cells expressing EGFP fusion constructs of wild type (WT) mVP40 and mutants. Cells were imaged 14 h post transfection. (**B**) COS-7 cells were quantified for the average plasma membrane localization for the respective constructs. Three independent experiments were performed for each construct. Error bars represent standard error of the mean. (C.I. = 95%). Scale bar = 10 µm.

COS-7 cells, which are from African green monkeys, have been used previously to study different aspects of filovirus research [33,34,35]. COS-7 cells were used in this study as they represent a viable cell model for VP40 related research [10] and monkeys can be infected with filoviruses. Live cell imaging of WT and the mVP40 hydrophobic loop region mutants 14 h post-transfection revealed that the single amino acid mutants of Phe^281^, Leu^283^ or Phe^286^ showed no detectable change in their ability to localize to the plasma membrane compared to WT mVP40 (Figure 2A,B). 

Plasma membrane localization of mVP40 was confirmed by using a plasma membrane marker, WGA-Alexa Fluor 647 and determining the overlap in fluorescence for EGFP-WT-mVP40 and WGA-Alexa Fluor 647 (Figure S1). In contrast, the monomeric mutant of mVP40 (T105R) abrogated the overlap in EGFP and WGA-Alexa Fluor 647 signals (Figure S1). Further, all single mutations displayed significant filamentous structures from the plasma membrane that resemble VLPs (Figure 2) akin to WT mVP40. Next, we prepared mutations to Phe^281^ and Phe^286^ residues (here onwards—double mutant) and a triple mutation of the Phe^281^, Leu^283^ and Phe^286^ residues (here onwards—triple mutant), which produced an abnormal phenotype compared to WT mVP40 and single mutations. Expression of double and triple mutants led to accumulation of GFP signal in intracellular bright spots that looked like “stalled-vesicles’ (Figure 2). Quantitative analysis of all aforementioned mutations demonstrated a significant reduction in plasma membrane localization of the double and triple mutations (Figure 2B).

**Figure 3:**
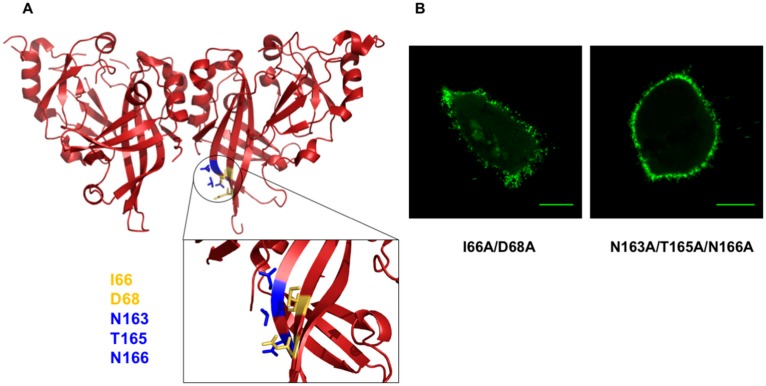
Multiple amino acid mutations to the mVP40 N-terminal domain (NTD )region adjacent to the C-terminal domain (CTD) hydrophobic loop failed to cause changes in plasma membrane localization compared to WT mVP40. (**A**) NTD region of mVP40 where the multiple amino acid mutants were made. (**B**) Cellular localization of the I66A/D68A and N163A/T165A/N166A mutant proteins following 14 h post-transfection in COS-7 cells. Scale bar = 10 µm.

To determine the selectivity of double or triple mutations in altering mVP40 plasma membrane localization, we prepared a double and triple mutation in the mVP40 NTD. These mutants were I66A/D68A and N163A/T165A/N166A (See Figure 3), which are part of the anti-parallel beta sheet structure near the bottom of the mVP40 NTD as shown in Figure 3A. In contrast to the double and triple mutations of the CTD discussed above, NTD mutations failed to appreciably alter plasma membrane localization or their ability to form filamentous structures from the plasma membrane (Figure 3B). Therefore, the abnormal “stalled-vesicles” morphology observed for the CTD double and triple mutants were considered significant in the context of mVP40 plasma membrane localization. 

### 3.3. Transmission Electron Microscopy

**Figure 4:**
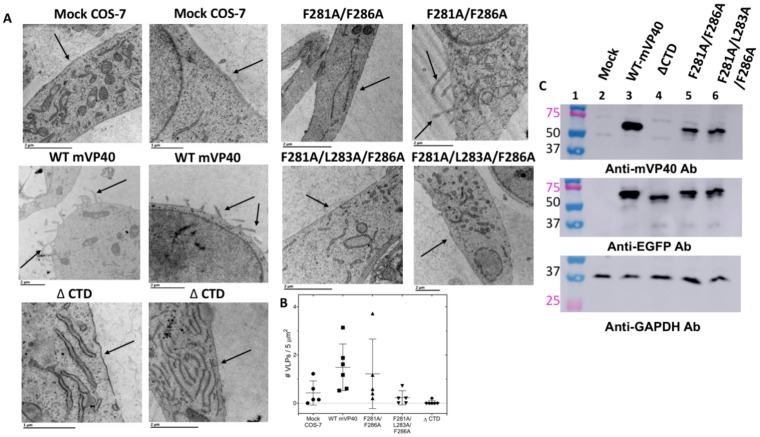
TEM and heterologous expression test of mVP40 and hydrophobic mutants. (**A**) Morphology of cells expressing WT mVP40 or mutant proteins. TEM images of COS-7 cells transfected with EGFP fusion constructs of WT-mVP40, F281A/F286A, F281A/L283A/F286A, or the CTD deletion. Arrows point to regions of the plasma membrane were formation or absence of pre-VLPs is evident for each respective construct. (**B**) To estimate the number of VLPs per area, the area of each cell was measured and the number of pre-VLPs (* only counted VLPs visibly connected to the plasma membrane surface) were counted. In the graph, values are reported as mean ± SD where a Brown-Forsythe and Welch ANOVA was then performed with multiple comparisons (compared to WT mVP40). *p* = 0.047 for CTD and *p* = 0.076 for F281A/L283A/L286A. (**C**) Expression of WT mVP40 and mutant proteins in COS-7 cells. Cell lysates collected from cells that were mock transfected (lane 2), expressing WT-mV40 (lane 3), or mutant mVP40 proteins (lanes 4–6) and were assessed by SDS-PAGE electrophoresis and western blot analysis by using anti-mVP40 antibody, anti-EGFP antibody and a control anti-GAPDH antibody. A ladder with molecular weight labeled was included in lane 1.

TEM was implemented to observe the morphology of the plasma membrane of cells expressing either mVP40 or the double and the triple mutants. From TEM micrographs, WT mVP40 expressing cells harbored filamentous structures emanating from the plasma membrane consistent with VLP formation (Figure 4A). In contrast, the double and the triple mutants observed a decrease in the amounts of these filamentous structures at the plasma membrane (Figure 4A). Although in a few cells VLP like structures were detectable for the double mutant (See left panel of the mutant in Figure 4A). Quantification of VLPs per µm^2^ from the TEM images indicated a decrease in the VLPs per area of cell plasma membrane for the double and triple mutant (Figure 4B). To recapitulate the results of the double and/or triple mutant, a CTD deletion mutant was prepared at residue Leu^201^ in mVP40. Similar to the double and triple mutation, the CTD deletion mutant also abrogated the formation of VLPs (Figure 4A,B).

To assess if the mutations affect the stability or the expression level of mVP40, we carried out a heterologous expression test. EGFP tagged mVP40 or mutants were expressed in COS-7 cells and the cell lysates were extracted and analyzed for protein expression by western blot. When a polyclonal mVP40 antibody was used, the double and triple mutant were detectable but the CTD domain deletion mutant protein expression was not detected suggesting the antibody binding epitope is in the CTD (Figure 4B). However, the CTD deletion was detectable when using an anti-EGFP antibody (Figure 4B). Compared to WT-mVP40, all the mutants showed slightly lower overall protein expression, which could be due to reduced stability, increased targeting for degradation, or the inability of the mutant proteins to undergo proper oligomerization beyond the dimer.

### 3.4. Assessment of WT mVP40 Dimer Structure and Lipid Binding Properties with Mutation of the CTD Hydrophobic Patch

**Figure 5:**
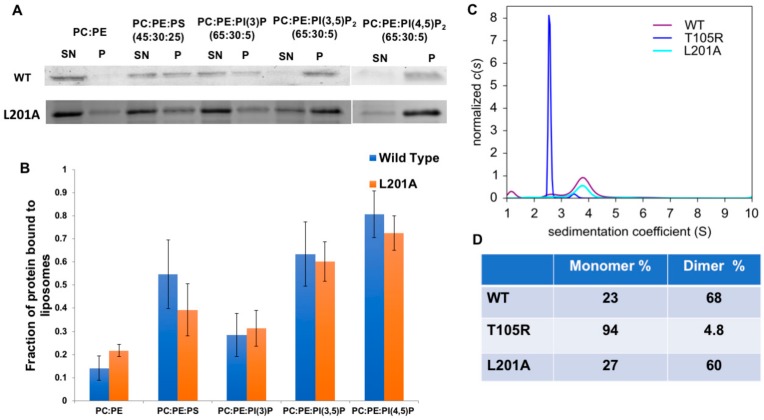
Biophysical properties of the L201A mutant are similar to WT mVP40 including anionic membrane binding properties and dimeric state in solution. (**A**) SDS-PAGE of supernatant (SN) and pellet (P) fractions collected from MLV sedimentation assays on lipid compositions and ratios appearing above the images for WT and L201A proteins. (**B**) Quantification of MLV sedimentation assays. The data represent the average of three sedimentation assays with error bars representing the standard error of the mean. (**C**) mVP40 protein size distribution was obtained through fitting data from sedimentation velocity experiments to continuous distribution c(s) Lamm equation model. (**D**) Monomer and dimer percentages of WT mVP40 (WT), T105R and L201A mutants obtained from sedimentation velocity experiments.

In order to rule out changes to mVP40 lipid binding or dimerization for mutation of the hydrophobic patch, we examined the role of the Leu^201^ residue of mVP40 which aligns with Leu^213^ of eVP40. Previous studies indicated that mutation of Leu^213^ in eVP40 limited plasma membrane localization and VLP formation as well as extensive VP40 oligomerization [19,20,21]. Leu^201^ also makes up part of the hydrophobic pocket with Phe^281^, Leu^283^, and Phe^286^. Thus, L201A was expressed and purified from bacteria and compared to mVP40 with respect to lipid-binding and its monomer/dimer ratio. An in vitro lipid binding assay using multilamellar vesicles (MLVs) was employed as it has been used to examine mVP40 lipid binding selectivity previously [8]. The L201A mutant showed similar membrane association properties to WT mVP40 indicating the mutation has not affected its membrane interaction properties (Figure 5A,B). Here, L201A and WT mVP40 associated similarly with vesicles containing anionic lipids such as PS, PI(3)P, PI(3,5)P_2_ or PI(4,5)P_2_. mVP40 and L201A were also identified to be predominantly dimeric in solution from sedimentation velocity experiments using analytical ultracentrifugation (AUC) (Figure 5C,D) as previously found [8]. WT and L201A were 68% and 60% dimeric, respectively, whereas a mutation of the dimer interface (T105R) caused mVP40 to be mostly monomeric (94%) in solution [8] (Figure 5C,D).

### 3.5. Investigation of WT mVP40 Co-Expression with mVP40 Hydrophobic Mutations

**Figure 6:**
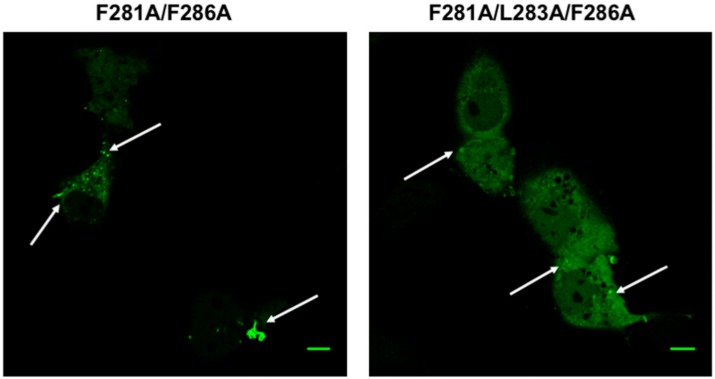
Co-expression of WT mVP40 failed to rescue the plasma membrane localization of hydrophobic loop mutants. COS-7 cells co-transfected with WT mVP40 (hemagglutinin (HA) tagged, 0.5 µg plasmid) and EGFP-mVP40 mutants (0.5 µg plasmid) imaged 14 hrs post-transfection. Scale Bar = 10 um.

We hypothesize that the abnormal phenotype we observed for the double and triple mutants was due to a disruption of mVP40 membrane trafficking rather than a structural distortion caused by the mutations because the mutations were located mainly within a small loop region and L201A retained membrane binding properties and the dimeric state. We next hypothesized that this phenotype may be reversed by allowing mutant monomers to dimerize with WT monomers by co-transfecting double or triple mutants along with WT mVP40. The strategy of WT VP40 co-expression with VP40 mutations [36] or fluorescently tagged VP40 [37] has previously been employed to examine VP40 trafficking or VLP formation, respectively. However, co-transfection with WT mVP40 failed to rescue the mutant phenotype and facilitate the trafficking of the mutants to the plasma membrane (Figure 6). The double mutant, however, exhibited a different morphology compared to imaging experiments where WT mVP40 was not present, where the double mutant exhibited more of a “stalled vesicle” morphology than that of the triple mutant, which exhibited a more diffuse expression pattern (Figure 6). This “stalled vesicle “morphology was seen in WT co-transfected triple mutant expressing cells as well but to a lesser degree. These results suggest the WT monomers dimerized with the double mutant monomers and may further proceed in the mVP40 membrane trafficking pathway compared to a dimer made of two double mutant monomers. It also strongly suggests that two functional monomers of mVP40 are needed to mediate host interactions and/or full trafficking to the plasma membrane. An alternative explanation could be that mVP40 WT dimers and double mutant dimers oligomerize further into higher ordered structures before they are able to interact with the plasma membrane. Nonetheless, the defective dimer results in oligomers that are incapable of forming a crucial host protein interaction that helps to facilitate the movement of mVP40 to the plasma membrane.

### 3.6. Molecular Dynamics Simulations of mVP40 and eVP40

**Figure 7:**
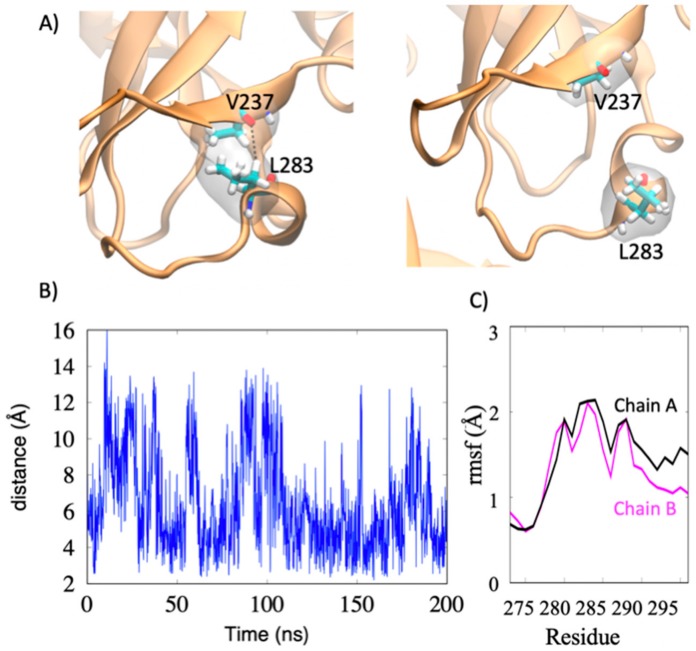
Flexibility of the mVP40 hydrophobic residues throughout the molecular dynamics simulation time. (**A**) Flexibility of the loop segment involving Leu^283^. (**B**) The distance between Val^237^-Leu^283^ over the course of the simulation. (**C**) Root-mean-square fluctuations of the residues in the loop segment (Chain A and B refer to the two VP40 protomers in the dimer structure).

As shown in Figure 1B, mVP40 loop residues Leu^201^, Phe^281^, Leu^283^, and Phe^286^ form a hydrophobic core similar to the hydrophobic core previously examined in eVP40 (Leu^213^, Leu^289^, Ile^293^, Leu^295^, and Val^298^). Molecular dynamics studies were performed of the mVP40 and eVP40 dimer to compare the dynamics of these two hydrophobic regions. In eVP40, the loop residues Ile^293^ and Leu^295^ display large flexibility (Supporting Information Movie S1). Interestingly, these residues, along with Val^298^ were found previously to insert into the PM [19,20]. In contrast to eVP40, mVP40 has a short alpha helix segment in the hydrophobic loop region. As a result, the residues Leu^283^, Phe^286^ (in the alpha-helix) and Phe^281^ (in the loop) are more restricted in mVP40 compared to the eVP40 residues Ile^293^ and Leu^295^, both in a more flexible loop (Movie S1), suggesting possible differences in cellular roles such as plasma membrane binding or interaction with host proteins. As shown in Figure 7, the flexible segment from residues 276–293 includes a helical turn involving Leu^283^. A transient hydrophobic interaction between Leu^283^ and Val^237^ coupled with the loop-flexibility gives large fluctuations in the Val^237^-Leu^283^ distance.

## 4. Discussion

mVP40, like its cousin eVP40, is the most abundant protein in the viral particle, forms the viral matrix layer, and provides structural stability to the viral particle [8,38]. Both eVP40 and mVP40 bind via electrostatic interactions to phospholipid membranes that contain anionic lipids [8,10,11,12,14,17,18,19,20]. These interactions are mediated by clusters of positively charged amino acids, known as the basic patch, at the CTD of these proteins [8,9,14]. From the crystal structures of eVP40 and mVP40 it was evident that the basic patches of their CTDs harbor some differences with respect to the mVP40 basic patch being flat and extended as well as more enriched in cationic residues compared to that of eVP40. The structural changes observed in the basic patches of eVP40 and mVP40 are consistent with their different selectivity for anionic membranes [10,11,14].

Apart from the basic patch, the CTD of eVP40 also contains a hydrophobic loop (Ile^293^, Leu^295^ and Val^298^) that mediates interactions with the plasma membrane through insertion into the hydrocarbon layer [19,20]. From our analysis of the crystal structure of mVP40 and eVP40, we found a loop region with hydrophobic character that is also present on mVP40. However, unlike eVP40, the mVP40 hydrophobic loop did not insert into the plasma membrane to form hydrophobic interactions [12]. However, the conservation of hydrophobic character as well as the orientation of this CTD loop region suggested a possible alternative function in mVP40 trafficking or plasma membrane mediated assembly.

In this study we sought to understand the role of hydrophobic loop region in mVP40. From our data, we demonstrated that multiple mutations to the hydrophobic loop resulted in abrogation of the plasma membrane inner leaflet localization characteristic of mVP40 assembly. We also showed that co-expression of WT mVP40 failed to restore mutants’ localization to the plasma membrane but produced a morphology where the mutant proteins appeared to be accumulating at intracellular membrane structures. This suggests that the double and triple mutants are still capable of interactions and their failure to appear at the plasma membrane could be due to mutant proteins failing to form critical host protein interactions necessary for membrane trafficking. Alternatively, the mVP40 hydrophobic loop may be important for the formation of higher ordered oligomers of the dimers where the double or triple mutants fail to undergo further oligomerization in order to assemble mVP40 filaments. Instead, perhaps the double and triple mutants assemble into dysfunctional/unordered oligomers that amass intracellularly.

**Figure 8:**
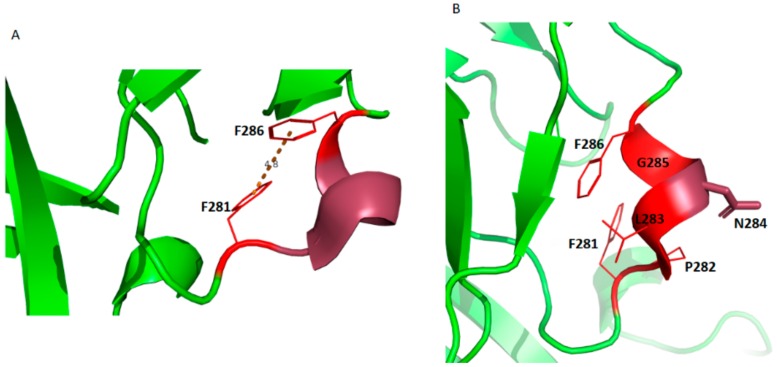
The mVP40 CTD hydrophobic loop may provide an interface for self-assembly or host protein interactions. (**A**) The two Phe residues present in the loop may form a T-shaped hydrophobic stacking interaction that may stabilize the loop’s orientation. The average distance between the aromatic rings of the two Phe residues is 4.8 Å. (**B**) Based on the outward orientation of the side chain of Asn^284^ , it is possible that Asn^284^ participates in key interactions (H-bonding) with a host protein or is involved in self-assembly.

Examining the orientation of Phe^281^ and Phe^286^, it is possible they may form hydrophobic stacking (T -shaped) interactions that could hold this hydrophobic loop at a particular orientation (Figure 8). This is also consistent with the lack of flexibility of this region in the MD simulations (Movie S1).

However, the contribution of this stacking interaction to the stability of this region should be minimal because point mutations of either Phe residue failed to prevent plasma membrane localization of the mutant protein. Based on the data we gathered, it is reasonable to hypothesize that the CTD hydrophobic loop region may be an interface for host protein interaction (with Asn^284^ being a key interacting residue as it has its side chain solvent exposed) or a self-assembly interface.

The Leu^213^ residue of eVP40 which was previously reported by McCarthy et al. to be important for plasma localization of the protein as an alanine substitution at the 213 position resulted in an altered cellular localization [21]. The Leu^213^ and the surrounding residues were identified as a bridging region between the NTD and CTD of eVP40 and as a result authors suggested this region to be important for overall structure and self-assembly. Therefore, we sought to understand how this hydrophobic residue may contribute to the overall stability and role of the hydrophobic patch. L201A exhibited similar lipid binding and dimer properties as WT mVP40 indicating these hydrophobic mutants may lack plasma membrane localization but it is unlikely due to reduction in membrane affinity or dimer formation. The ability of the L201A mutant to form VLPs remains to be examined.

**Figure 9:**
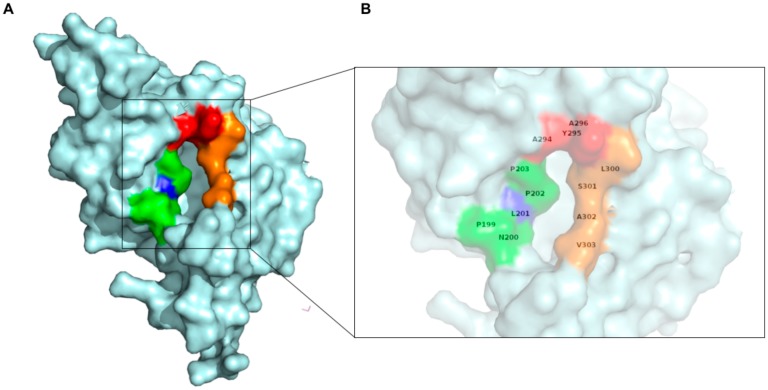
mVP40 Leu^201^ residue may be part of a hydrophobic pocket that can interact with a host factor. (**A**) mVP40 monomer highlighting Leu^201^ (in blue) and neighboring residues forming a hydrophobic pocket. (in green residues 199–203, in red residues 294–296, and in orange residues 300–303) (**B**) A zoomed image of the hydrophobic pocket that includes Leu^201^.

Based on our results, we hypothesize the Leu^201^ residue contributes to the stability of the mVP40 hydrophobic patch and likely has a role in mVP40 self-assembly and/or host protein interactions. Leu^201^, along with neighboring residues (residues: Pro^199^, Asn^200^, Pro^202^, Pro^203^, Leu^300^, Ser^301^, Ala^302^, and Val^303^) forms a hydrophobic pocket structure (Figure 9). This pocket may function as a binding site to a host factor or it could be a self-assembly interface. mVP40 is also thought to undergo a conformational change that facilitates oligomerization similar to the eVP40 protein, where the CTD detaches from the NTD to expose a hydrophobic oligomerization surface containing Trp^83^ [8] that aids in forming an oligomeric structure. Based on the orientation of Trp^83^ and Leu^201^ and their neighboring residues, movement of Leu^201^ and its adjacent residues following membrane association would result in an exposure of Trp^83^. Therefore, it is possible that the Leu^201^ residue has an important role in mVP40 self-assembly.

## 5. Conclusions

In conclusion, our findings indicate the mVP40 CTD hydrophobic loop consisting of residues Phe^281^, Leu^283^, and Phe^286^ is important for intracellular trafficking and plasma membrane localization of mVP40. The mutational study also emphasized the necessity of the fine balance between the affinities of mVP40 to different host factors (or itself) that may allow mVP40 to associate and dissociate from them during the course of intracellular trafficking pathways to reach the plasma membrane. This study also underscores the subtle but different roles of CTD residues in eVP40 and mVP40 with respect to interactions with host factors or lipids during the viral replication cycle.

## Supplementary Materials

The following figure and movie are available online at https://www.mdpi.com/1999-4915/12/4/482/s1, Figure S1: Plasma membrane localization of mVP40. (**A**) COS-7 cells expressing EGFP-WT-mVP40 and were stained with a plasma membrane (PM) stain (WGA-Alexa Fluor 647) 14 h post-transfection and imaged on a confocal microscope. White lines were overlaid onto the image to indicate where plot profile analysis was performed. (**B**) A histogram of fluorescence signals of PM stain (pink line) and WT-mVP40 (green line) from plot profile analysis performed in ImageJ. (**C**) COS-7 cells expressing EGFP-T105R-mVP40 were stained with a PM stain (WGA-Alexa Fluor 647) 14 h post-transfection and imaged on a confocal microscope. White lines were overlaid onto the image to indicate where plot profile analysis was performed. (**D**) A histogram of fluorescence signals of PM stain (pink line) and T105R-mVP40 (green line) from plot profile analysis was performed in ImageJ. (**E**) Quantification of PM localization of EGFP-WT-mVP40 using ImageJ. Automatic thresholding is applied to an initial image (original image and thresholding image) to create a ROI outlining the cellular periphery (plasma membrane ROI) and of the intracellular space (intracellular ROI) to create a ROI which selects the plasma membrane (final ROI). ROIs are indicated by white lines. Scale bar = 10 µm. WT: wild-type; mVP40: Marburg virus viral protein 40; PM: plasma membrane; WGA: wheat germ agglutinin; ROI: region of interest. Movie S1. Molecular dynamics simulations of the flexibility of the hydrophobic patch in eVP40 and mVP40.
