# Supplementary figures and images for: Mutation of Hydrophobic Residues in the C-Terminal Domain of the Marburg Virus Matrix Protein VP40 Disrupts Trafficking to the Plasma Membrane
Source: Viruses. 2020 Apr 24;12(4):482. doi: 10.3390/v12040482 (PMC7232359; doi:10.3390/v12040482)

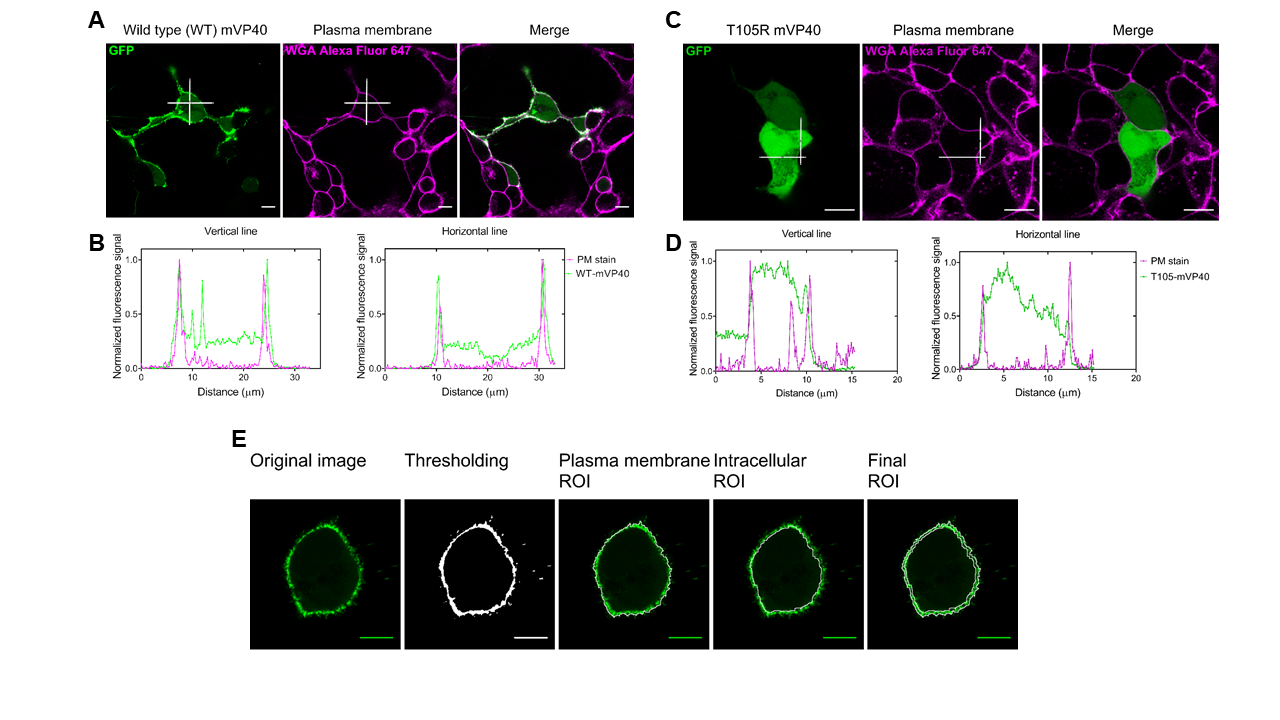

Supplement: Supplementary file 1 [file viruses-12-00482-s001.zip › viruses-746069-suppl/figure s1.tif]
